# Supplementary material for: SARS-CoV-2 Spike Mutations, L452R, T478K, E484Q and P681R, in the Second Wave of COVID-19 in Maharashtra, India
Source: Microorganisms. 2021 Jul 20;9(7):1542. doi: 10.3390/microorganisms9071542 (PMC8307577; doi:10.3390/microorganisms9071542)
Supplement: Supplementary file 1 [file microorganisms-09-01542-s001.zip › microorganisms-1226235-SI.pdf]

Supplementary Figures & Tables

Figure S1: The distribution of predominant PangoLIN lineages in the labelled districts of Maharashtra. The size of the pie charts is proportional to the number of genome sequences from the particular district.

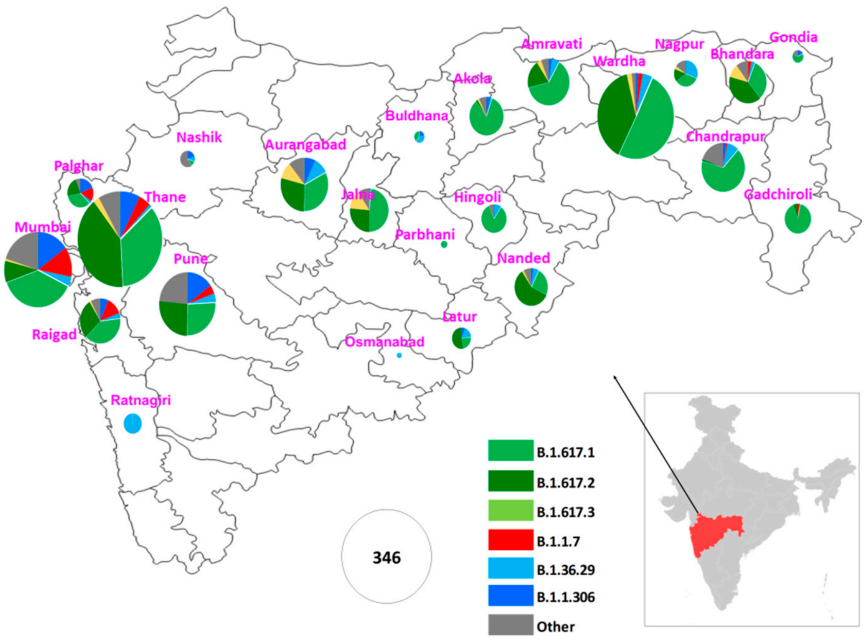

Figure S2: Heat map of major mutations in the different proteins of the predominant lineages from November, 2020 to May, 2021

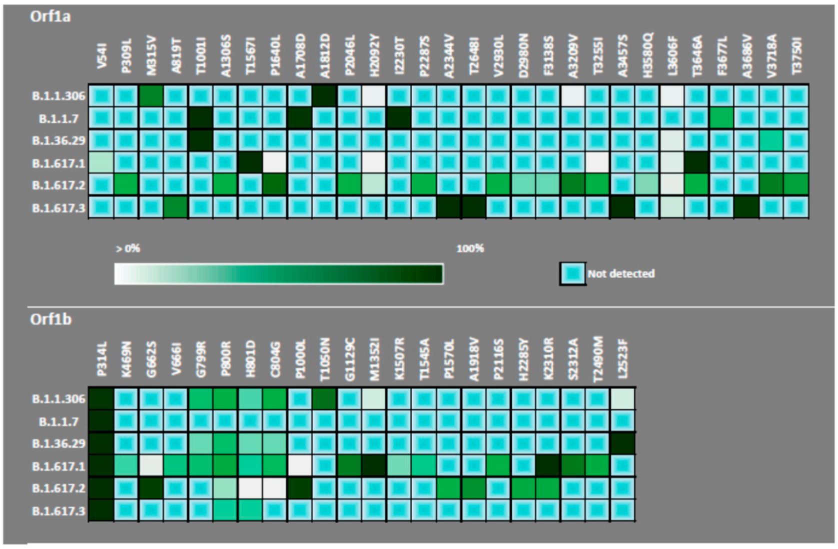

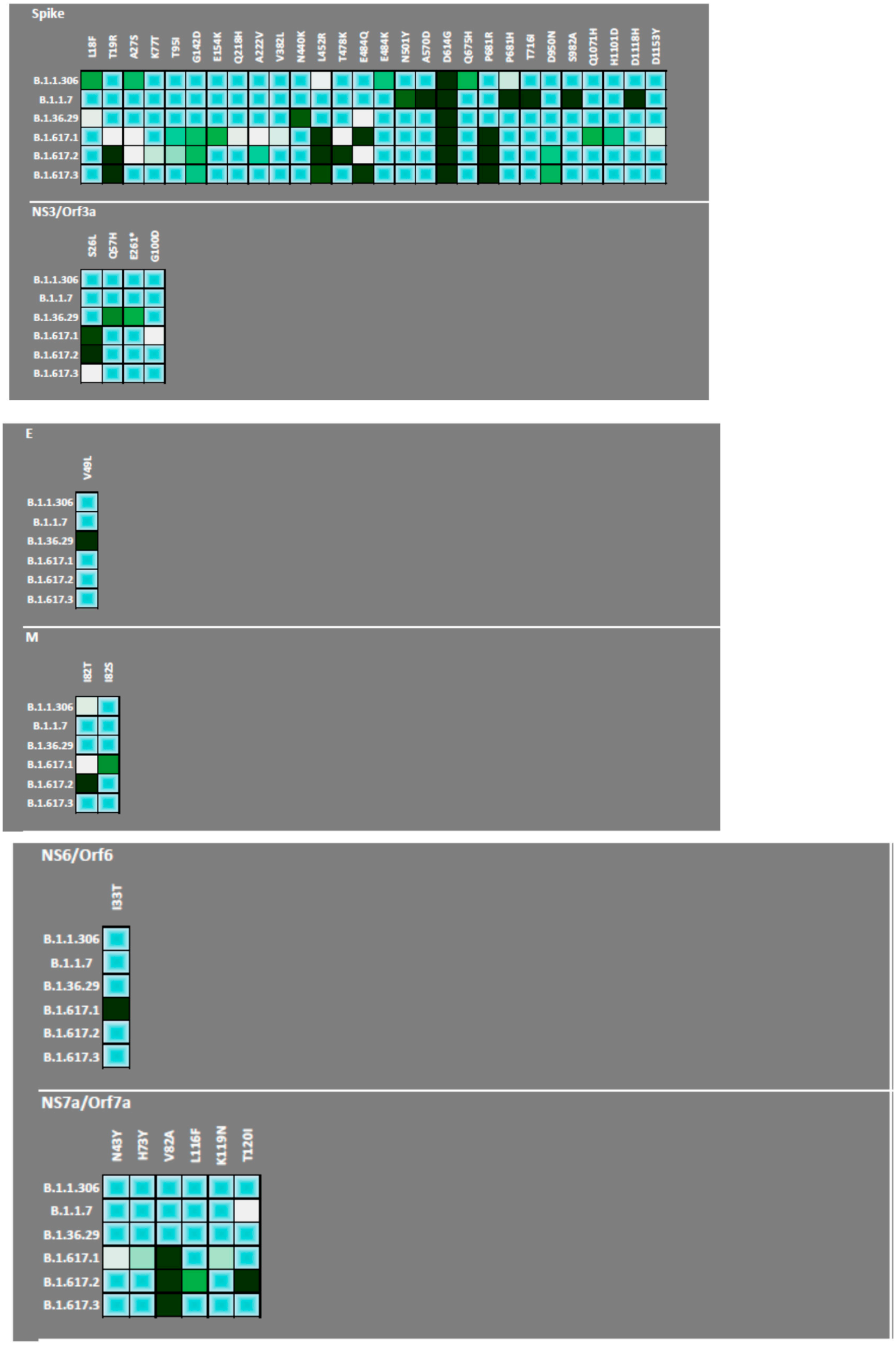
Spike
L18F
T19R
A27S
K77T
T95I
G142D
E154K
Q218H
A222V
V382L
N440K
L452R
T478K
E484Q
E484K
N501Y
A570D
D614G
Q675H
P681R
P681H
T716I
D950N
S982A
Q1071H
H1101D
D1118H
D1153Y
B.1.1.306
B.1.1.7
B.1.36.29
B.1.617.1
B.1.617.2
B.1.617.3
NS3/Orf3a
S26L
Q57H
E261*
G100D
E
V49L
M
I82T
I82S
NS6/Orf6
I33T
NS7a/Orf7a
N43Y
H73Y
V82A
L116F
K119N
T120I

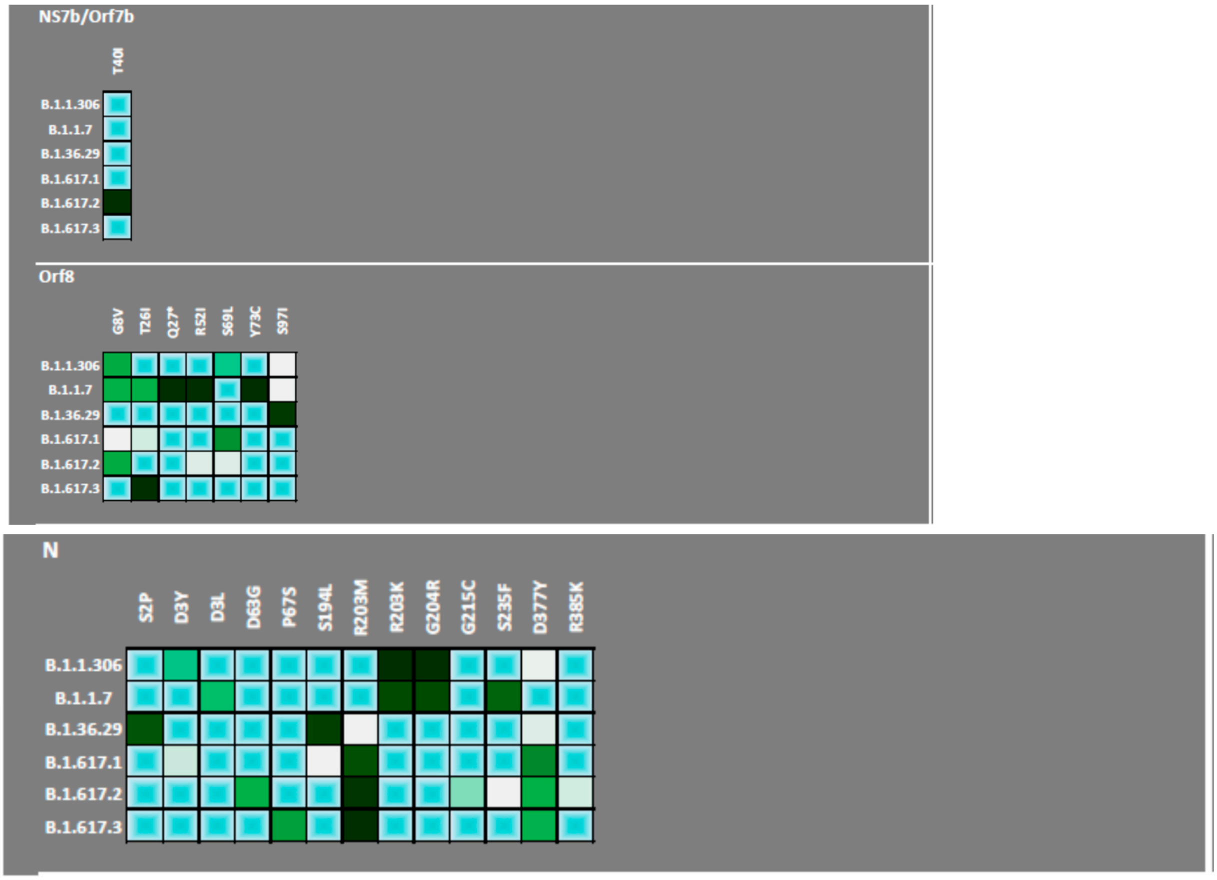

Figure S3: Structural complexes of RBD-ACE2 and RBD-Fab. **A**. RBD - ACE2 (PDB Id-6LZG), **B**. RBD - mAb REGN10933(PDB Id-6XDG) **C**. RBD - mAb P2B-2F6(7bwj)

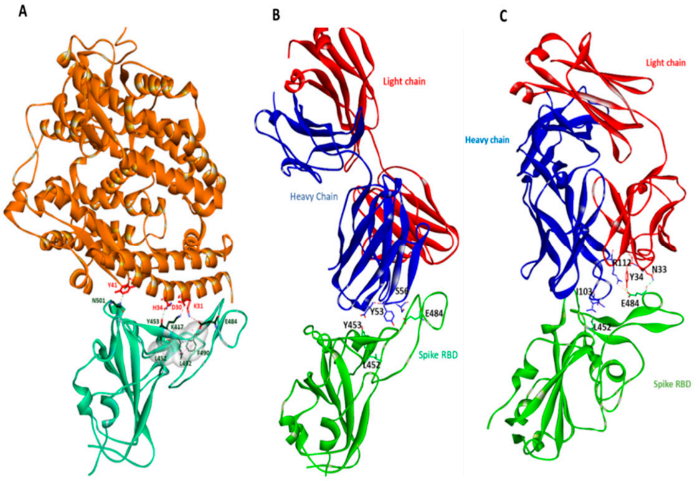

Figure S4: Modeling of the SARS-CoV-2 spike protein (with the furin cleavage motif) and effect of P681R mutation (A) Complete spike protein with the residues in the furin cleavage site labelled (B) Wild-type strain with P681 (C) Mutant strain with P681R

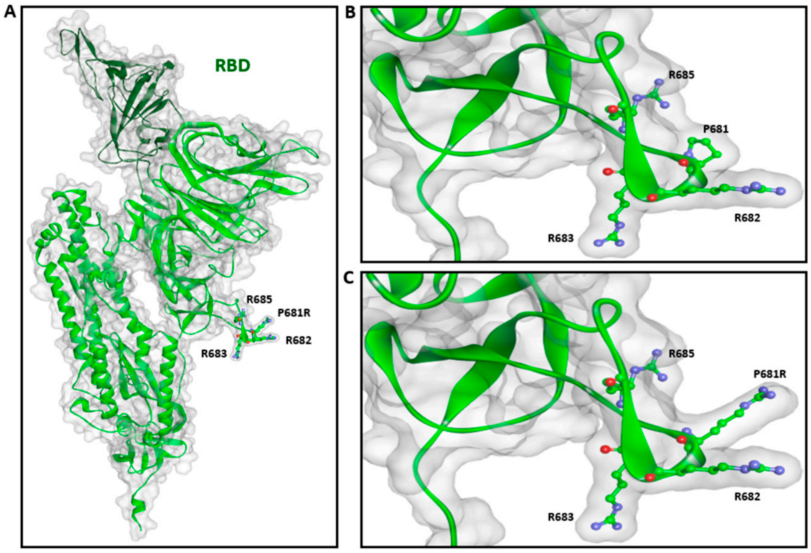

Figure S5: Temporal trend of major lineages (top twelve) in India based on sequences from GISAID (As of June 6, 2021) from November, 2020 to May, 2021 (A) India inclusive of Maharashtra (n= 9495) (B) India excluding Maharashtra (n=6949).

(A)

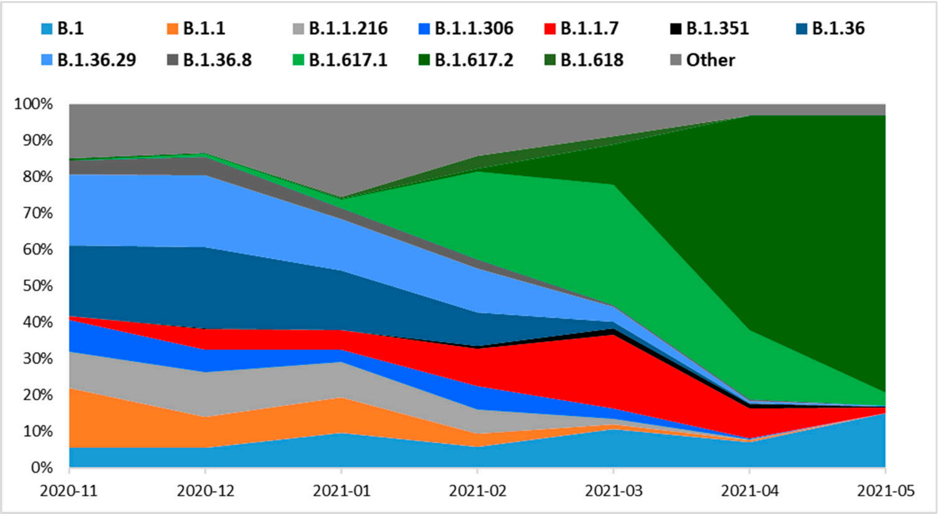

(B)

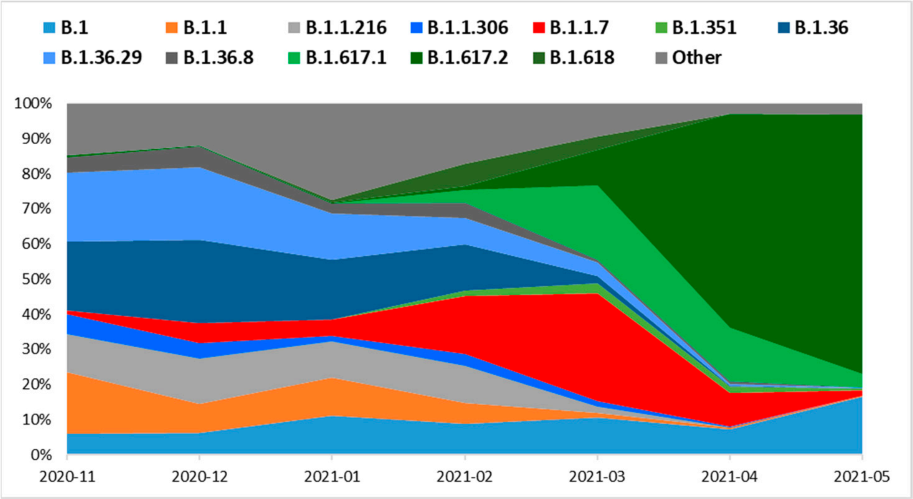

Supplementary Table S1

Supplementary Table S1: Intra and inter-molecular interactions of the wild-type and mutant spike RBD of SARS-CoV-2 with ACE2. The bonds that are made bold in the wild-type indicate those that are disrupted in the mutant strain while the bonds that are bold in the mutant indicate those that are additional when compared to the wild-type strain. Mutant1 indicates the strain of lineage B.1.617.1 and mutant2 indicates a strain of lineage B.1.617.2.

Spike RBD with ACE2 (6LZG.pdb) (wild-type) Interactions			
**Sr. No.**	**RBD-Wild Type**	**ACE2**	**Type**
1	K417	D30	Hydrogen Bond; Electrostatic
**2**	**E484**	**K31**	**Electrostatic**
3	A475	S19	Hydrogen Bond
4	N487	Q24	Hydrogen Bond
5	T500	Y41	Hydrogen Bond
6	G496	K353	Hydrogen Bond
7	Y449	D38	Hydrogen Bond
8	Y449	Q42	Hydrogen Bond
9	N487	Y83	Hydrogen Bond
10	Q493	E35	Hydrogen Bond
11	Q498	Q42	Hydrogen Bond
12	Q498	Q42	Hydrogen Bond
13	G502	K353	Hydrogen Bond
14	Y453	H34	Hydrogen Bond
15	F486	Y83	Hydrogen Bond
16	N501	Y41	Hydrogen Bond

17	L455	H34	Hydrophobic
18	F486	Y83	Hydrophobic
19	Y505	K353	Hydrophobic
20	F486	M82	Hydrophobic
21	Y489	K31	Hydrophobic
22	Y505	K353	Hydrophobic
**23**	**L452-L492**	**-**	**Hydrophobic**
24	F490-L492	-	Hydrophobic
**Spike RBD with ACE2 (6LZG.pdb) (L452R & E484Q Mutant1)**			
**Interactions**			
**Sr. No.**	**RBD-Mutant Type**	**ACE2**	**Type**
1	K417	D30	Hydrogen Bond; Electrostatic
2	A475	S19	Hydrogen Bond
3	N487	Q24	Hydrogen Bond
4	T500	Y41	Hydrogen Bond
5	G496	K353	Hydrogen Bond
6	Y449	D38	Hydrogen Bond
7	Y449	Q42	Hydrogen Bond
8	N487	Y83	Hydrogen Bond
9	Q493	E35	Hydrogen Bond
10	Q498	Q42	Hydrogen Bond
11	Q498	Q42	Hydrogen Bond
12	G502	K353	Hydrogen Bond
13	Y453	H34	Hydrogen Bond
14	F486	Y83	Hydrogen Bond
15	N501	Y41	Hydrogen Bond
16	L455	H34	Hydrophobic
17	F486	Y83	Hydrophobic
18	Y505	K353	Hydrophobic
19	F486	M82	Hydrophobic
20	Y489	K31	Hydrophobic
21	Y505	K353	Hydrophobic
22	F490-L492	-	Hydrophobic
**Spike RBD with ACE2 (6LZG.pdb) (L452R & T478K Mutant2)**			
**Interactions**			
1	K417	D30	Hydrogen Bond; Electrostatic
2	A475	S19	Hydrogen Bond
3	N487	Q24	Hydrogen Bond
4	T500	Y41	Hydrogen Bond
5	G496	K353	Hydrogen Bond
6	Y449	D38	Hydrogen Bond
7	Y449	Q42	Hydrogen Bond
8	N487	Y83	Hydrogen Bond

9	Q493	E35	Hydrogen Bond
10	Q498	Q42	Hydrogen Bond
11	Q498	Q42	Hydrogen Bond
12	G502	K353	Hydrogen Bond
13	Y453	H34	Hydrogen Bond
14	F486	Y83	Hydrogen Bond
15	N501	Y41	Hydrogen Bond
16	L455	H34	Hydrophobic
17	F486	Y83	Hydrophobic
18	Y505	K353	Hydrophobic
19	F486	M82	Hydrophobic
20	Y489	K31	Hydrophobic
21	Y505	K353	Hydrophobic
22	F490-L492	-	Hydrophobic
**23**	**K478-F486**	**-**	**Hydrogen Bond**
**24**	**K478-F486**	**-**	**Hydrogen Bond**
**25**	**K478-F486**	**-**	**Hydrogen Bond**

Supplementary Table S2: Intermolecular interaction of neutralizing antibodies with wild-type and mutant spike RBD of SARS-CoV-2

A	**Spike RBD with Antibody REGN10933 (Wild-type)**		
	**Interactions**		
	**RBD**	**Antibody**	**Type**
1	GLU484	H: TYR53	Hydrogen Bond
		H:SER56	
2	LYS417	H:ASP31	Hydrogen Bond
		H:THR28	
3	PHE486	L: LEU94	Hydrophobic
		H: ARG100	Electrostatic
		H:TYR59	Hydrogen bond
4	TYR489	H: TYR53	Hydrophobic
	TYR489	H:TYR33	Hydrogen Bond
5	TYR453	H:ASP31	Hydrogen Bond
6	LYS417	H:ASP31	Hydrogen Bond
7	ALA475	H:MET104	Hydrophobic bond
B	**Spike RBD with Antibody REGN10933 (Mutant)**		
	**Interactions**		
	**RBD**	**Antibody**	**Type**

1	LYS417	H:ASP31	Hydrogen Bond
		H: THR28	
2	TYR453	H :ASP31	Hydrogen Bond
3	PHE486	H: ARG100	Hydrogen Bond
		L: LEU94	
		H:TYR59	
4	ALA475	H:MET104	Hydrophobic bond
5	TYR489	H:TYR53	Hydrophobic
6	GLN484	H:SER56	Hydrogen bond
7	CYS488	H:TYR33	Hydrogen bond
C	**Spike RBD with Antibody P2B-2F6(Wild-type)**		
	**Interactions**		
	**RBD**	**Antibody**	**Type**
1	LEU452	H: ILE103	Hydrophobic
		H:VAL105	
2	GLU484	L: ASN33	Hydrogen bond
		L:TYR34	
3	GLU484	H:ARG112	Hydrogen bond; Electrostatic
4	PHE490	H: VAL106	Hydrophobic
		H:PRO107	
5	TYR449	H: SER31	Hydrogen bond
6	ASN450	H: SER30	Hydrogen bond
		H:SER31	
7	GLY447	H: TYR27	Hydrogen bond
D	**Spike RBD with Antibody P2B-2F6 A(Mutant)**		
	**Interactions**		
	**RBD**	**Antibody**	**Type**
1	GLN484	L: ASN33	Hydrogen bond
		L: TYR34	
2	ASN450	H: SER30	Hydrogen bond
		H:SER31	
3	PHE490	H: VAL106	Hydrophobic
		H: PRO107	

Supplementary Table S3: Major spike protein mutations defining the B.1.617 lineages and proposed effect on structural aspects

S.No.	Mutations	Lineage/s	Proposed Effect on structural aspects	Available Reference/s
1.	E484Q	B.1.617.1, B.1.617.3	Decreased binding to select mAb[#]	32,33
2.	L452R	B.1.617.1, B.1.617.2, B.1.617.3	Decreased binding to select mAb[#]	26, 32, 34, 35, 36
3.	L452R and E484Q	B.1.617.1, B.1.617.3	Enhanced ACE2[$] binding	24, 32, 36, 37,
4.	L452R and T478K	B.1.617.2	Enhanced ACE2[$] binding	38
5.	P681R	B.1.617.1, B.1.617.2, B.1.617.3	Enhanced binding to furin	24

[#] Monoclonal antibody
[$]Angiotensin converting enzyme
